# *Mycoplasma genitalium* Biofilms Contain Poly-GlcNAc and Contribute to Antibiotic Resistance

**DOI:** 10.3389/fmicb.2020.585524

**Published:** 2020-10-27

**Authors:** James M. Daubenspeck, Arthur H. Totten, Jason Needham, Monica Feng, Mitchell F. Balish, T. Prescott Atkinson, Kevin Dybvig

**Affiliations:** ^1^Department of Pediatrics, University of Alabama at Birmingham, Birmingham, AL, United States; ^2^Department of Microbiology, University of Alabama at Birmingham, Birmingham, AL, United States; ^3^Department of Microbiology, Miami University, Oxford, OH, United States; ^4^Department of Genetics, University of Alabama at Birmingham, Birmingham, AL, United States

**Keywords:** *Mycoplasma genitalium*, mollicutes, biofilm, antibiotic resistance, poly-GlcNAc

## Abstract

*Mycoplasma genitalium* is an important etiologic agent of non-gonococcal urethritis (NGU), known for chronicity and multidrug resistance, in which biofilms may play an integral role. In some bacterial species capable of forming biofilms, extracellular polymeric substances (EPS) composed of poly-*N*-acetylglucosamine (PNAG) are a crucial component of the matrix. Monosaccharide analysis of *M. genitalium* strains revealed high abundance of GlcNAc, suggesting a biofilm-specific EPS. Chromatograms also showed high concentrations of galactose and glucose as observed in other mycoplasma species. Fluorescence microscopy of *M. genitalium* biofilms utilizing fluor-coupled lectins revealed differential staining of biofilm structures. Scanning electron microscopy (SEM) showed increasing maturation over time of bacterial “towers” seen in biofilm development. As seen with *Mycoplasma pneumoniae*, organisms within fully mature *M. genitalium* biofilms exhibited loss of cell polarization. Bacteria associated with disrupted biofilms exhibited decreased dose-dependent viability after treatment with antibiotics compared to bacteria with intact biofilms. In addition, growth index analysis demonstrated decreases in metabolism in cultures with disrupted biofilms with antibiotic treatment. Taken together, these data suggest that *M. genitalium* biofilms are a contributing factor in antibiotic resistance.

## Introduction

Mycoplasmas are small, obligate bacterial parasites that are host-restricted with a genome typically <1 Mbp. They have no cell wall and require host-derived cholesterol. With a genome of 577 to 590 kb, *M. genitalium* has the smallest genome of any naturally occurring prokaryote known that is capable of independent replication (Su and Baseman, 1990). *M. genitalium* is a sexually transmitted pathogen and a causative agent of acute and chronic non-gonococcal urethritis in men (Tully et al., 1981; Taylor-Robinson and Jensen, 2011; Taylor-Robinson, 2017). There is evidence that it also causes cervicitis and pelvic inflammatory disease in women (Gnanadurai and Fifer, 2019). *M. genitalium* is rarely culturable and can take >6 months to adapt to growth *in vitro*. In estimates, 1–2% of the population is positive for *M. genitalium* in the urogenital tract, while in sexual health clinics this can rise to as high as 38% of all patients (Van Der Pol et al., 2020). Studies have also recently suggested that asymptomatic carriage rates (10–12%) may be as high as other sexually transmitted infection (STI)-associated pathogens (Taylor-Robinson and Jensen, 2011; Gaydos et al., 2019; Van Der Pol et al., 2020). Given the relatively high prevalence of *M. genitalium* in STI clinics (Gottesman et al., 2017; Chra et al., 2018; Gaydos et al., 2019) and its propensity for development of resistance to multiple antimicrobial agents (Dionne-Odom et al., 2018; Xiao et al., 2018, 2019; Bachmann et al., 2020), new measures for control of infection are urgently needed. In order to institute control measures, better understanding of the mechanisms of antibiotic resistance and virulence associated with biofilms by *M. genitalium* will be important.

Bacterial biofilms consist of cells encased within an extracellular matrix composed of an aggregation of polysaccharides, polypeptides, nucleic acids, and lipids that when combined form a bacterial shelter. The bacteria within the biofilm secrete polymeric substances to construct the matrix, a defining characteristic of bacterial biofilms (Hall-Stoodley et al., 2004). Whether these molecules are synthesized by the bacteria or pirated from the host (mycoplasmas do both), the role biofilms play in protecting the bacteria is critical. Several *Mycoplasma* species including *Mycoplasma pneumoniae*, a human pathogen, (Simmons et al., 2013; Simmons and Dybvig, 2015; Feng et al., 2018) and *Mycoplasma pulmonis*, a rodent pathogen, form biofilms *in vitro* and *in vivo* (Simmons and Dybvig, 2009). Similarly, *Ureaplasma* species have been shown to form biofilms *in vitro*, suggesting that the ability to form higher complexity bacterial structures is found across the class *Mollicutes* (Garcia-Castillo et al., 2008; Pandelidis et al., 2013). An attractive explanation for the chronicity associated with mycoplasmal disease is that the bacteria form these structures *in vivo*, shielding the organism from host immune components and therapeutic agents (Hall-Stoodley et al., 2004; Feng et al., 2020).

*Mycoplasma genitalium* infections are typically treated with drugs from three main classes: macrolides, fluoroquinolones, and tetracyclines. Despite adequate *in vitro* efficacy of tetracyclines, treatment failure is high in patients (∼70%) in spite of the lack of reported *tetM* or other known resistance mechanisms (Peyriere et al., 2018). Current data from the United States and international locations suggest that macrolide resistance rates range between 40 and 80% in various patient populations (Gesink et al., 2016; Getman et al., 2016; Anderson et al., 2017; Trembizki et al., 2017; Dionne-Odom et al., 2018). Recent reports in Alabama have shown the macrolide resistance rate in heterosexual couples was approximately 60%, with the quinolone resistance rate at about 11% (Xiao et al., 2019). Strikingly, macrolide resistance rates were between 70 and 80% in HIV-positive men, and quinolone resistance rates were in the 20–40% range (Dionne-Odom et al., 2018; Xiao et al., 2019). Insufficient penetration of antibiotics into the biofilm matrix, poor patient compliance, and inadequate dosing are all additional possible contributions to the rising occurrence of clinically untreatable infections.

In this study, we show that *M. genitalium* forms biofilms *in vitro* as evidenced by a marked increase in exopolysaccharide production by surface-associated bacteria as well as electron microscopic images demonstrating the progressive production of towers and the development of a strikingly thickened, “wooly” appearance of the bacteria over time. Furthermore, we identify PNAG as a component of the biofilm. Finally, we show that the *M. genitalium* biofilm decreases antibiotic access, leading to a lower level exposure and increased survival, thus increasing the risk for the development of resistance. This work highlights the importance of *M. genitalium* biofilms and may open new avenues for treatment of *M. genitalium* infections.

## Materials and Methods

### Bacterial Strains and Growth Conditions

*Mycoplasma genitalium* strain G37 was previously purchased from the American Type Culture Collection (33530). Clinical isolate UAB BHM-1A (UAB 73697) was the generous gift of the UAB Diagnostic Mycoplasma Laboratory (Birmingham, AL, United States). Both *M. genitalium* strains were cultured in SP4 medium at 37°C for up to 14–30 days until a pH change indicative of growth was observed (Xiao et al., 2018). Bacteria were harvested and stored in aliquots at −80°C. For carbohydrate analysis, cultures were harvested at 10,000 × *g* for 30 min at 4°C, and the resulting pellets were washed twice in phosphate-buffered saline (PBS, Gibco, Thermo Fisher Scientific). Bacteria were suspended in PBS and stored at −20°C until GC-MS analysis.

### Scanning Electron Microscopy (SEM) of *M. genitalium* Biofilms

*Mycoplasma genitalium* strain G37 was inoculated in triplicate (input CFU of ∼1 × 10^6^) and grown into biofilms on 13-mm glass coverslips that were submerged in SP4 broth in 24-well tissue culture plates. Coverslips were assessed for biofilm formation by SEM at 48-h intervals over a period of 144 h. Samples were processed as previously described by Relich and Balish (2011). Briefly, SP4 broth was gently aspirated and replaced with a fixative solution of 1.5% glutaraldehyde, 1% formaldehyde, and 0.1 M sodium cacodylate (pH 7.2) for 30 min at room temperature. Samples were then dehydrated in increasing concentrations of ethanol (25–100%, v/v), critical-point dried, and gold sputter-coated as described by Hatchel et al. (2006). *M. genitalium* biofilm samples were visualized using a Zeiss Supra 35 VP FEG scanning electron microscope (Zeiss) at the Miami University Center for Advanced Microscopy and Imaging.

### Biofilm Growth and Analysis

Biofilm analysis was carried out for *M. genitalium* as was previously done for other *Mycoplasma* species (Daubenspeck et al., 2009; Simmons et al., 2013). In brief, *M. genitalium* strains G37 and UAB BHM-1A were grown in T25 flasks (BD Biosciences) with 20 mL SP4. To harvest non-adhered bacteria and bacteria associated with biofilms, the medium containing non-adhered bacteria was decanted, and flasks washed gently twice in PBS. Washes were decanted and 5 mL of water was added to each flask. Cell scrapers (Corning) were utilized to disrupt adhered portion of culture on flask surface. Resultant aggregate mass was spun at 2,000 × *g* at room temperature for 10 min. The original medium containing the non-adhered bacteria was spun at 20,000 × *g* for 10 min to collect bacteria not associated with adhered fraction. The pellet from the adhered fraction of the culture was considered to contain biofilm and associated bacteria, and the pellet from the SP4 medium was considered a non-adhered fraction. Both pellets were washed × 2 in PBS and suspensions were frozen at −20°C until carbohydrate analysis.

*Mycoplasma genitalium* strain G37 was diluted in SP4 from frozen stock to about 1 × 10^5^ CFU/mL and 1 mL of the suspension was then added to each well of 12-well flat-bottom culture plates under aseptic conditions. Plates were incubated at 37°C until the medium turned orange and towers were visible by 100x light microscopy, indicating sufficient bacterial growth. Upon processing, medium was carefully removed, neutral buffered formalin (Thermo Fisher Scientific) was added to each well at a final 4% concentration, and plates were incubated at 4°C overnight for fixation. After fixation, wells were washed twice in PBS and the washes discarded. The fixed cells were stored at 4°C in PBS until processing for staining and imaging.

A panel of lectins (EY Laboratories) was selected for biofilm analysis based on GC and MS results: ConA (binding specificity per EY laboratories: α-methyl-mannopyranoside >α-D-mannose>α-D-glucose >α-*N*-acetyl-D-glucosamine), GS-I (binding specificity: α-D-galactoside and α-linked galactose oligosaccharides), GS-II (binding specificity: *N*-acetyl-D-glucosamine) and PNA (binding specificity: lactose >β-D-galactose). *M. genitalium* biofilms were stained with the selected lectins by permeabilizing with 0.3% Triton X-100 and incubating with FITC-conjugated lectins (1:100 in 1x PBS + 5% BSA) for 1 h. Biofilms were then mounted using ProLong Glass Antifade Mountant with or without NucBlue (Invitrogen). Images were taken using a Keyence BZ-X810 fluorescence microscope with at least eight images captured per treatment. Images were quantified using the “raster 3.0-2” package in R studio using R version 3.0 by taking the geometric mean of the image pixels that were both above the background intensity, as determined by the negative control, and below saturation.

### Gas Chromatography/Mass Spectrometry (GC-MS)

Samples of biofilm pellets were desiccated and subjected to methanolysis with 400 μl of acidic methanol at 80°C for 12 h to generate the methyl glycosides. Re-*N*-acetylation was carried out with the addition of 25 μl of acetyl chloride and 25 μl of pyridine in 150 μl of methanol for 30 min. The samples were volatilized by the addition of 50 μl of trimethylsilyl (Thermo Fisher Scientific). Samples were analyzed on a Varian GC-MS in the electron ionization mode. The monosaccharide composition was determined by comparison with known standards from Sigma-Aldrich.

#### Exopolysaccharide Gel Extraction

Cultures were grown in SP4 medium attached in T-75 tissue culture flasks to late log phase. Cultures were scraped and harvested by centrifugation. Harvested biofilms were washed three times in PBS, lysed by sonication and then digested with DNase and RNase overnight. The culture was then digested with proteinase K overnight. The samples were dialyzed against water for 5000 volumes in a 2000-Da dialysis cassette, the sample volume was reduced under vacuum and then run on a 4–12% gradient *Tris-*glycine SDS-PAGE gel. The relevant material was excised out of the gel and extracted by repeated dehydration with 50% acetonitrile. The fractions were pooled and analyzed by GC-MS.

#### Staining of SDS-PAGE Gels for Carbohydrate

4–15% gradient SDS-PAGE gels were utilized for this analysis. Pro-Q^®^ Emerald 300, a periodic acid-based stain (Thermo Fisher Scientific), was used to stain for glycosylation. Standard laboratory reagents and techniques were utilized for Coomassie Brilliant Blue staining.

### Flow Cytometry

Bacterial cultures were prepared as previously described (see section “Bacterial Strains and Growth Conditions”) for G37 and UAB BHM-1A. Upon color change, 0.5 mL aliquots of medium containing non-adhered bacteria/small aggregates were collected from the cultures and centrifuged at 20,000 × *g* for 5 min at 4°C. The medium was discarded and pellets suspended in PBS. Following PBS wash, fluorescently labeled lectins (EY Laboratories) were added to the suspensions at 20 μg/mL. After incubation for 20 min at room temperature, suspensions were washed three times in PBS. Cells were then suspended in PBS and analyzed for flow cytometric profiles by flow cytometry (C6 Accuri, BD Biosciences). The resulting data were visualized and analyzed by FloJo v. 10 (BD Biosciences) and Novocyte software (ACEA Biosciences, Inc., United States).

### Antibiotic Treatment of Bacterial Biofilms

G37 cultures were prepared as previously described in 1 mL SP4 in 12-well tissue culture plates. Upon color change, the plates were inspected to ensure biofilm structure formation by visualization of bacterial aggregates and towers by light microscopy under 100X magnification. To disrupt biofilms, the plates were floated in a 1.9L ultrasonic water-bath sonicator (Thermo Fisher Scientific) for 10 min at low power to break bacterial aggregates, as described in our previous studies on bacterial disaggregation (Totten et al., 2017). Confirmation of disruption was carried out pre- and post-sonication by light microscopy. Disrupted and non-disrupted cultures were incubated at 37°C for an additional hour to allow cell membrane recovery from the sonication process, as described previously (Totten et al., 2017). After incubation, three dilutions of antibiotics (High, Medium and Low) (Sigma Aldrich) diluted in SP4 broth at 2x final concentration (μg/mL) were added: erythromycin (4, 0.4, and 0.04), levofloxacin (10, 1, and 0.1), and doxycycline (2.5, 0.25, and 0.025).

These concentrations were chosen based on the known minimum inhibitory concentration of *M. genitalium* strain G37 as determined by the UAB Diagnostic Mycoplasma Laboratory. The medium concentration of antibiotic represents the minimum inhibitory concentration for each respective antibiotic [Personal communications, (Carroll et al., 2019)]. Untreated control wells were supplemented with an equal volume of SP4. To control for minute ethanol concentrations in Ery preparations, 50 μl of ethanol was added per 2 mL of SP4 for “EtOH control” wells. Biofilms were then incubated for 2 days until harvesting for imaging or growth index analysis as described previously (Feng et al., 2018; Totten et al., 2019). In brief, the medium was analyzed by determining OD 430/OD 560 for each condition and data were plotted as described.

For tracking of antibiotic stress on *M. genitalium* biofilms, the methionine analog L-homopropargylglycine (HPG, Click Chemistry Tools) and the thymine analog 5-ethynyl-2′-deoxyuridine (EdU, Thermo Fisher Scientific) were stored in DMSO at 1 mM concentrations at −80°C until use. After plate sonication and 1 h incubation at 37°C, 1 μl of EdU or 3 μl of HPG were added to each mL of culture medium. EdU, which tracks DNA synthesis, was added to control wells as well as those treated with Levo, and HPG, which tracks protein biogenesis, was added to control wells as well as those treated with Doxy and Ery. Fixation and washing were carried out as outlined for previously described fluorescent biofilm imaging. EdU and HPG conjugations were performed following permeabilization using a click-it reaction (20 μM AF488 azide, 2 mM copper sulfate, 10 mM sodium ascorbate in 1x PBS) for 1 h. Images were taken using the Keyence BZ-X810 fluorescence microscope as described above under Biofilm Growth and Analysis.

### Statistical Analyses

For data analysis, sample sizes of <3 were examined for significant differences by either unpaired student *T*-test (parametric data), or Mann-Whitney *U* test (non-parametric data). For experimental analyses with groups >3, Two-way ANOVA was carried out with Sidak’s multiple comparisons post-test. For multiple post-test comparisons, multiplicity-adjusted *P*-values were utilized to correct for multiple cross-comparisons. For visual/graphical representation, the following were used as representative symbols: ^∗^ for *p* < 0.05, ^∗∗^ for *p* < 0.01, ^∗∗∗^ for *p* < 0.001, and ^****^ for *p* < 0.0001. For data that were not significantly different, NS stood for “not significantly different,” and ND stood for “not determined.” GraphPad Prism v. 8.4 software (GraphPad, Inc.) was used for calculation of statistical significance and graphical visualization.

## Results

### *Mycoplasma genitalium* Forms a Biofilm *in vitro*

**FIGURE 1:**
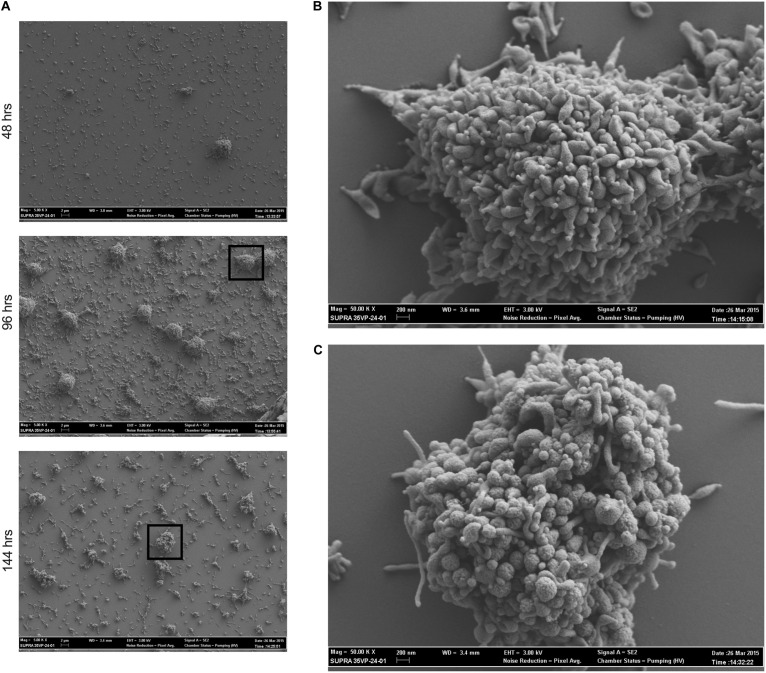
*Mycoplasma genitalium* forms biofilms *in vitro*. Scanning electron microscopy time course of *M. genitalium* biofilms of strain G37. **(A)** G37 was grown in SP4 and imaged at 48, 96, and 144 h post-inoculation at resolutions of 5,000×. **(B)** 50,000× view of 96 h time point of G37. **(C)** 50,000× view of 144 h time point of G37. Experimental observations repeated in at least two independent experiments per time point.

To determine whether *M. genitalium* forms biofilms that resemble those of other bacteria (Peyriere et al., 2018), strain G37 was grown on glass cover slips and examined by SEM and imaged at 48, 96, and 144 h post-inoculation (Figure 1). The resulting biofilm towers shared some general similarities with those of the closely related species *M. pneumoniae*, but also exhibited some differences. *M. genitalium* cells appeared densely packed even in early-stage (48-h) towers, and as they grew, they acquired a “hairy” or “wooly” appearance, similar to *M. pneumoniae* (Feng et al., 2018), likely associated with accumulation of EPS over time. At this early stage of growth, the *M. genitalium* cells on the surfaces of the towers had an overall less elongated appearance than *M. pneumoniae*, consistent with the absence of a trailing filament in individual *M. genitalium* cells (Hatchel and Balish, 2008). Despite this more compact appearance, cells nonetheless tended to become even more rounded as tower growth continued to 144 h, in parallel to *M. pneumoniae* but at a somewhat slower pace (Feng et al., 2018). Adjacent to the towers, individual adherent *M. genitalium* cells as well as aggregates of cells that were much smaller than towers were present, but possibly to a lesser extent than for *M. pneumoniae* (Feng et al., 2018).

### Monosaccharide Analysis of *Mycoplasma genitalium* Biofilms

**FIGURE 2:**
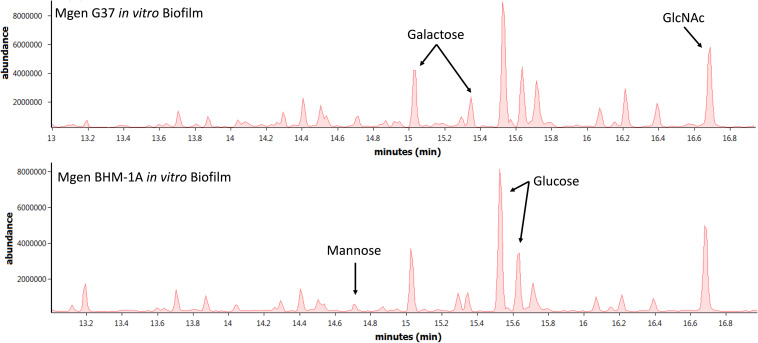
Monosaccharide analysis of *Mycoplasma genitalium*. Representative GC-MS spectra of strains G37 and UAB BHM-1A. Experimental observations repeated in triplicate. Arrows indicate peaks of interest on GC-MS spectra.

**FIGURE 3:**
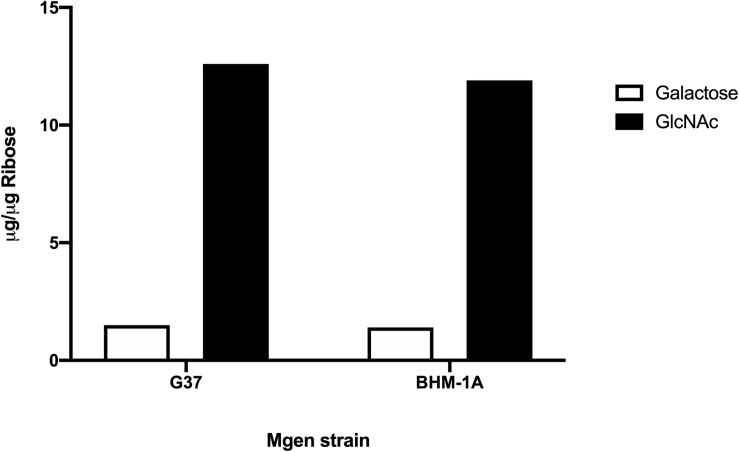
Area under the curve of GlcNAc and galactose from strains G37 and UAB BHM-1A in SP4. Values were obtained by comparison to standard curves utilizing known standards. Normalization was carried out using ribose as an internal standard.

Biofilms of *M. genitalium* type strain G37 and a clinical isolate, UAB BHM-1A, were harvested and subjected to monosaccharide analysis using GC-MS (Figure 2). Chromatograms from both strains exhibited similar profiles, with glucose, galactose, and *N*-acetylglucosamine (GlcNAc) being predominant. Unattached cultures of *M. genitalium* strain G37 showed a similar monosaccharide profile (data not shown). Comparison of GC samples to a GlcNAc standard curve demonstrated a high concentration of the carbohydrate present in the *M. genitalium* biofilm samples (Figure 3). The large excess of GlcNAc is not apparent in the chromatograms seen in Figure 2 because GC characteristically exhibits low sensitivity for GlcNAc. Initial testing of G37 and UAB BHM-1A showed large differences in growth rates with UAB BHM-1A taking much longer (2–3 weeks) for mature biofilm formation. For this reason and because their biofilm composition appears similar by GC-MS, G37 was selected for further experimentation.

### *Mycoplasma genitalium* Biofilm Exopolysaccharide Is Composed Primarily of GlcNAc

**FIGURE 4:**
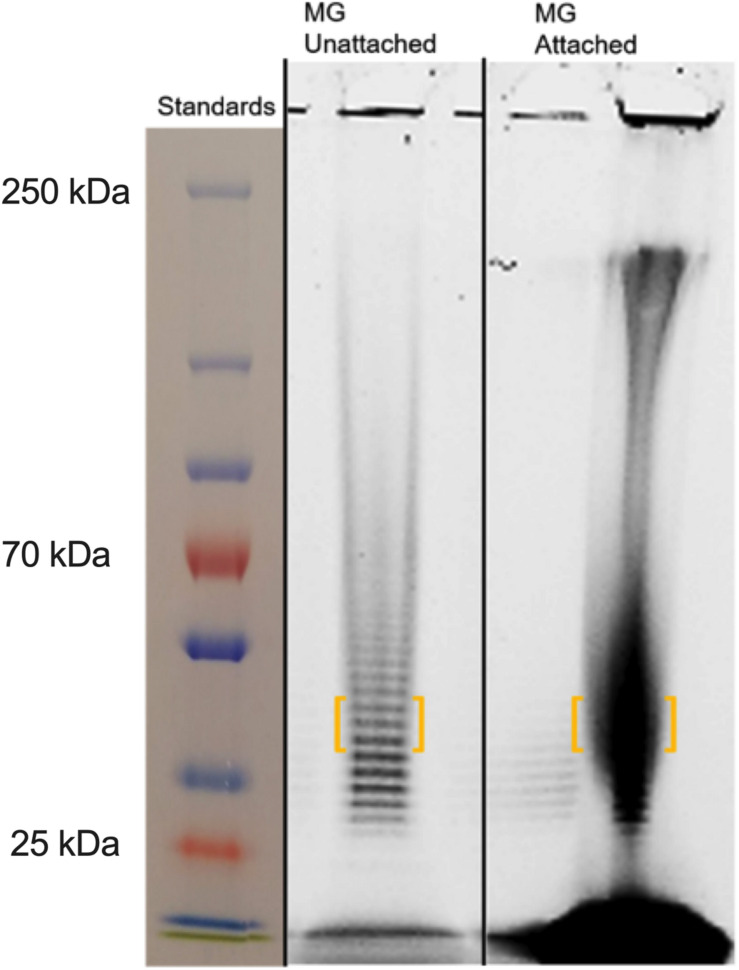
SDS-PAGE of *M. genitalium* strains G37 and BHM-1A harvested from biofilms. Mated gels were run and then stained with either Coomassie or Emerald 300 (glyco). Experimental observations repeated in duplicate. Orange brackets outline portions of lanes that were excised for further analysis by GC (see Figure 5).

**FIGURE 5:**
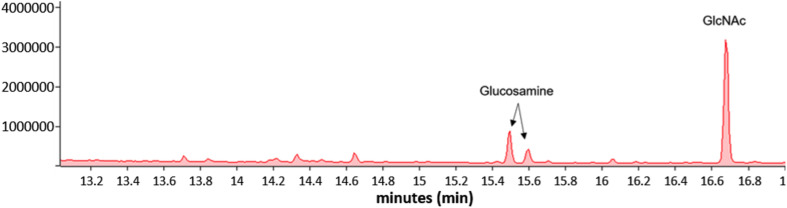
GC of the excised material from orange brackets from the “attached lane” in Figure 4 showing the presence of GlcNAc and glucosamine. The GC profile of the material excised from the “unattached” lane was essentially identical.

*Mycoplasma genitalium* type strain G37 was grown attached in tissue culture flasks or unattached in 50-ml conical tubes in SP4. The cultures were harvested, lysed and digested with DNase and RNase, followed by digestion with proteinase K. The samples were then dialyzed against water with a 2-kDa cutoff. Samples were analyzed by SDS-PAGE and stained with Emerald 300, a saccharide-specific glycostain (Figure 4). To verify the monosaccharide content of the *M. genitalium* exopolysaccharide, the staining material (highlighted by orange brackets in Figure 4) was excised and extracted. The GC chromatogram from the attached sample is shown in Figure 5. The chromatograms from the attached and unattached gels were essentially identical, so only the attached chromatogram is shown. There is a strong GlcNAc peak along with a residual glucosamine. There is evidence for a phosphorylated lipid. The GC chromatogram shown in Supplementary Figure S1 is the lipid region of the chromatogram shown in Figure 5. The MS signature shown in Supplementary Figure S2 is taken from the peak at 17.90 in Supplementary Figure S1 and shows consecutive losses of 14 m/z, a clear indication of a lipid.

### Glycomoieties Are Associated With Cellular and Biofilm Structure Formation in *Mycoplasma genitalium*

**FIGURE 6:**
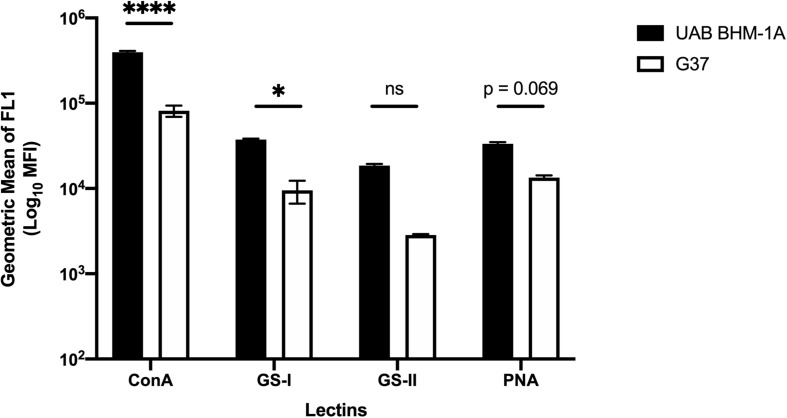
*Mycoplasma genitalium* carbohydrate production between strains. Flow cytometric measurement of lectin binding to unattached *M. genitalium* strains G37 and UAB BHM-1A grown in SP4. Shown is the net mean fluorescence intensity of bacteria bound by FITC-labeled lectins ConA, GS-I, GS-II, and PNA compared to unstained bacteria. Representative flow data from a minimum of two experiments. ^∗^*p* < 0.05 and ^****^p < 0.0001.

To examine the cell-associated extracellular matrix, *M. genitalium* cultures of type strain G37 and UAB BHM-1A were stained with fluorescein isothiocyanate (FITC)-labeled lectins and compared by flow cytometry. Lectins were selected based on the monosaccharide analysis in Figure 2 and availability. All four lectins (ConA, GS-I, GS-II, and PNA) showed significantly increased staining compared to unstained controls (Figure 6). Staining with ConA, unlike the other lectins, revealed a bimodal population distribution with both G37 and UAB BHM-1A (Figure 6). This population was enriched in the clinical isolate compared to the type strain, and overall lectin staining was significantly higher with UAB BHM-1A than G37 (Figure 6). These data taken together suggest potential strain-specific differences in polysaccharide production, which may reflect differences between lab-adapted and clinical isolates.

**FIGURE 7:**
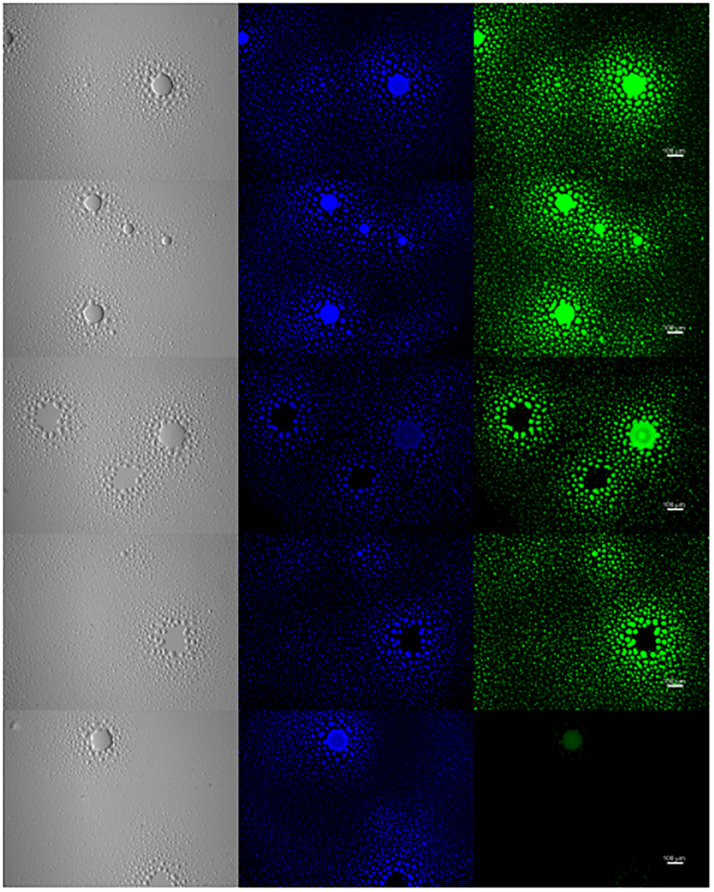
Exopolysaccharide is associated with *M. genitalium* biofilms. *M. genitalium* biofilms were imaged using DAPI (blue), and FITC-labeled lectins ConA, GS-I, GS-II and PNA (green). Shown are representative biofilm images from three independent experiments. Scale bar is 100 μm in size across all panels.

To further examine the presence of EPS in biofilm formation, *M. genitalium* strain G37 was cultured *in vitro* and examined by lectin-based immunofluorescence microscopy to assess the composition of biofilm structures. After 5–8 days, G37 biofilms were easily visible by light microscopy with some tower structures appearing larger than 100 μm (Figure 7). DNA staining with 4’,6-diamidino-2-phenylindole (DAPI) showed large aggregates of bacteria within the towers and also present around the base of the larger frameworks. The same panel of FITC-labeled lectins used to examine EPS associated with bacteria by flow cytometry (ConA, GS-I, GS-II, and PNA) was selected to analyze biofilm composition by fluorescence microscopy (Figure 7). Smaller bacterial aggregates stained with higher intensity by automated quantitative image analysis than larger structures with ConA and PNA, in agreement with flow cytometric data (data not shown). Staining of the smaller bacterial aggregates with GS-I and GS-II, which bind to α-D-galactoside/α-linked galactose oligosaccharides and GlcNAc, respectively, was reduced compared to the staining of towers.

### *Mycoplasma genitalium* Biofilms Increase Resistance to Antibiotic Exposure *in vitro*

*Mycoplasma genitalium* biofilms were tested to determine whether resistance to antibiotic exposure was increased compared to disrupted biofilms. G37 biofilms were grown *in vitro*, and then three doses (high, medium, and low) of doxycycline (Dox), levofloxacin (Levo) and erythromycin (Ery), representatives of the three main antibiotic classes used to treat clinical *M. genitalium* infections, were added to the cultures.

**FIGURE 8:**
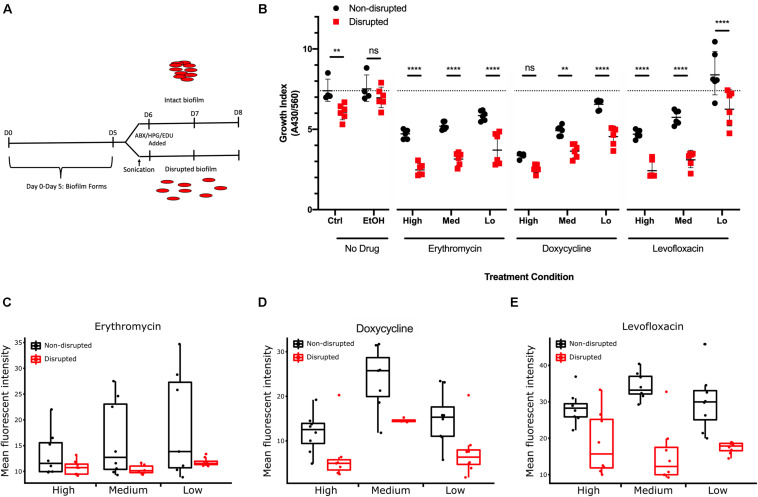
Effect of *M. genitalium* biofilms on antibiotic-mediated inhibition. **(A)** Eight-day time course schematic showing the incubation times, points of sonication, and addition of antibiotics, HPG, and EDU. **(B)** Metabolic index determined by spectrophotometry on the culture medium at 8 days (430/560 nm) from cultures with or without the indicated concentrations of antibiotics. Dotted line indicates the control group geometric mean growth index for comparative purposes of all groups. **(C–E)** Comparison of the effects of three concentrations of each antibiotic on cultures with disrupted and non-disrupted biofilms as determined by AF488 conjugated HPG **(C,D)** or EdU **(E)**. For each condition, ≥8 randomly selected images were analyzed for the geometric mean pixel intensity. Data are represented by box plot with the middle line representing the median intensity value, the box representing the upper and lower quartile range, and the whiskers indicating maximum 1.5 IQR. ^∗∗^*p* < 0.01 and ^****^p < 0.0001.

Cultures were treated with antibiotics for 48 h after biofilm disruption or mock treatment, and growth index analysis was undertaken to examine the effects of biofilm formation on resistance to antibiotic stress (Figure 8A). All three antibiotics caused a significant decrease in growth indices of G37 compared to untreated controls (Figure 8B). A dose-response curve was observed, indicating that decreasing antibiotic concentration resulted in increased *M. genitalium* growth index. Across all antibiotic concentrations tested except for the Levo low and Dox high, growth indices were significantly lower in disrupted biofilms compared to non-disrupted cultures.

As an independent measure of resistance to antibiotics, molecular methods for examining protein biogenesis and DNA synthesis (the mechanisms of action of the drugs Ery/Dox, and Levo, respectively) were undertaken. Labeling was done through incorporation of a methionine analog L-Homopropargylglycine (HPG) for tracking protein biogenesis or incorporation of a nucleoside analog of thymidine, 5-ethynyl-2′-deoxyuridine (EdU), for DNA synthesis tracking. HPG or EdU was added to the growth medium 24 h prior to fixation to track protein biogenesis and DNA synthesis rates, respectively. Incorporation of HPG and EdU decreased within *M. genitalium* biofilms with all antibiotics compared to control (no antibiotics) (Figures 8C–E and Supplementary Figure S3). With Ery and Dox treatment, a dose-dependent inhibition occurred in uptake of HPG (green, Alexa Fluor 488 (AF488) azide conjugation). Small-to-mid sized structures within the biofilms were greatly decreased in size and uniformity with antibiotic treatment while larger structures were preserved suggesting that more protection from antibiotics is offered by larger biofilm structures (Supplementary Figure S3). A dose-dependent decrease in uptake of EdU labeling with exposure to Levo also occurred indicating decreased DNA synthesis rates with increasing drug concentration (Supplementary Figure S3). Thus, the dose-related antibiotic-mediated inhibition of measures of bacterial growth, protein biogenesis and DNA synthesis rates is decreased for cells encased in larger intact biofilm structures (Figures 8C–E). These data taken together support the hypothesis that biofilms inherently increase antimicrobial resistance.

## Discussion

*Mycoplasma genitalium* is an important etiologic agent of several urogenital disorders including non-gonococcal urethritis, cervicitis, and pelvic inflammatory disease. Antibiotic resistance rates are increasing steadily, and many infections are clinically untreatable with currently approved antibacterial drug regimens in the United States. Our study represents the first thorough and mechanistic analysis of *M. genitalium* secreted EPS and its role in biofilms. Additionally, we present evidence of a role for biofilm in *M. genitalium* antibiotic resistance.

Carbohydrate research in mollicutes has been one of the more neglected fields compared to other bacterial species. However, all the components traditionally associated with *Firmicutes* are present in *Mycoplasmas*. A traditional capsule has been demonstrated to exist in several species, including *Mycoplasma mycoides* subsp. *capri*, which has capsule composed of furanose-galactan (Buttery and Plackett, 1960). Capsule has also been observed in *M. pulmonis* (Daubenspeck et al., 2009) and is thought to be present in *M. pneumoniae* (Simmons et al., 2013). Mycoplasmas post-translationally modify proteins, with two currently known modification systems in the genus. Data from *M. pneumoniae* suggest that galactose and GlcNAc are components of the same polysaccharide and that this polymer is essential for biofilm formation (Simmons et al., 2013).

The data presented here strongly suggest that *M. genitalium* has a polysaccharide composed of PNAG. Although PNAG is an adhesive polysaccharide associated with biofilms of many bacterial species (Lin et al., 2015; Roux et al., 2015), PNAG has not previously been identified in any species of mycoplasma. Given the close phylogenetic relationship between *M. genitalium* and *M. pneumoniae*, it is surprising that they produce different polysaccharides. There is no obvious pathway for GlcNAc biosynthesis in mycoplasmas. The identification of the genes required for PNAG synthesis would be an important area of study.

Biofilm formation in the closely related species *M. pneumoniae*, as well as *M. pulmonis* and the ureaplasmas, has been reported (Garcia-Castillo et al., 2008; Simmons and Dybvig, 2009, 2015; Pandelidis et al., 2013; Simmons et al., 2013; Feng et al., 2018). In the case of *M. pneumoniae*, attachment but not motility was necessary for biofilm formation (Feng et al., 2018). Biofilms have also been shown to form *in vivo* in other bacterial species and contribute directly to antibiotic resistance and persistence of infection in patients (Sharma et al., 2019). There are many reports of persistent detection of *M. genitalium* upward of 100 days after antibiotic therapy and clinical cure (Gnanadurai and Fifer, 2019). Data presented in our study show that *in vitro* bacterial growth may be impacted by antibiotic treatment with *M. genitalium* in intact biofilms better able to tolerate high levels of drug. Antibiotics slowed metabolism and incorporation of HPG and EdU but did not fully halt these processes. Disrupted biofilms compared to non-disrupted controls showed significantly larger antibiotic-dependent decreases in metabolic growth indices and in fluorescent markers of protein and DNA synthesis. Non-disrupted biofilm towers appeared largely unaffected by antibiotic treatment, suggesting that drugs that impair synthesis of or degrade EPS might synergize with antibiotic therapy in improving treatment efficacy. It remains to be seen how these *in vitro* studies may relate to the development of *in vivo* antibiotic resistance by *M. genitalium*.

Analogous to previous speculation regarding a role for towers in transmission of respiratory infections with *M. pneumoniae*, tower structure degradation or sloughing may be important for STI transmission by *M. genitalium* (Feng et al., 2018). As Feng et al. described, aggregates are more likely to serve as the foundation for tower formation, rather than single cells (Feng et al., 2018). Our microscopy analysis has shown that tower structures are prone to sloughing, unlike the smaller satellite structures, suggesting that this form of bacterial dissemination is similar between *M. pneumoniae* and *M. genitalium* (Feng et al., 2018). As the infectious unit is not currently established for either *M. pneumoniae* or *M. genitalium*, it is intriguing to conjecture that person-to-person spread may be through larger bacterial aggregates rather than single cells. It is even possible that *M. genitalium* cells are always aggregated and planktonic cells do not exist *in vivo*. *Vibrio cholerae* biofilms are more infectious than planktonic cells, and mycoplasma species require extensive disruption to acquire small aggregate sizes (Tamayo et al., 2010; Totten et al., 2017). There is some evidence that the initial steps of protection afforded by biofilms are operative during host-host transmission, supporting these hypotheses (Simmons and Dybvig, 2007).

Since its discovery in 1981, *M. genitalium* has been clearly described as a host cell-associated bacterium (Tully et al., 1981). Work over the last 30 years has been devoted to understanding potential intracellular associations with *M. genitalium* (Mernaugh et al., 1993; Ueno et al., 2008; McGowin et al., 2009) and *M. pneumoniae* (Yavlovich et al., 2004). Although previous studies have shown potential intracellular localization via extracellular gentamicin pulse treatment, these data are still debated (Ueno et al., 2008; McGowin et al., 2009). Previous microscopic analysis of *M. genitalium* has shown structures reminiscent of traditional biofilms, but little work was done to expand upon these observations (Mernaugh et al., 1993; Ueno et al., 2008). Our work is the first to describe biofilm-associated antibiotic resistance in *M. genitalium*. Additionally, our data provide the first substantive characterization of the extracellular matrix of *M. genitalium* and support a role for biofilm in the development of antibiotic resistance.

## Data Availability Statement

The original contributions presented in the study are included in the article/Supplementary Material, further inquiries can be directed to the corresponding author/s.

## Author Contributions

AT, JD, MB, KD, and TA designed and planned the initial experimentation, and interpreted initial experimental results. JN, MF, JD, and AT ran and interpreted the experiments and wrote the first manuscript draft. All authors contributed to drafting, interpreting, editing and finalizing the manuscript, and work described herein.

## Conflict of Interest

The authors declare that the research was conducted in the absence of any commercial or financial relationships that could be construed as a potential conflict of interest.

## Abbreviations

AF488: Alexa Fluor 488
ConA: concanavalin A
DAPI: 4 ′, 6-diamidino-2-phenylindole
Dox: doxycycline
EdU: 5-ethynyl-2 ′ -deoxyuridine
EPS: extracellular polymeric substances
Ery: erythromycin
FITC: fluorescein isothiocyanate
GC-MS: Gas chromatography–mass spectrometry
GlcNAc: N-acetylglucosamine
GS-I/-II: Griffonia simplicifolia I/-II
HIV: human immunodeficiency virus
HPG: L-homopropargylglycine
Levo: levofloxacin
*M. genitalium*: *Mycoplasma genitalium*
MSM: men who have sex with men
NGU: non-gonococcal urethritis
PBS: phosphate buffered saline
PNA: peanut agglutinin
PNAG: poly-N-acetylglucosamine
PTM: post-translational modification
SEM: scanning electron microscopy
STI: sexually transmitted infection.
