# Supplementary material for: Mycoplasma genitalium Biofilms Contain Poly-GlcNAc and Contribute to Antibiotic Resistance
Source: Front Microbiol. 2020 Oct 27;11:585524. doi: 10.3389/fmicb.2020.585524 (PMC7652822; doi:10.3389/fmicb.2020.585524)
Supplement: Supplementary file 1 [file Data_Sheet_1.docx]

Supplementary Material

**
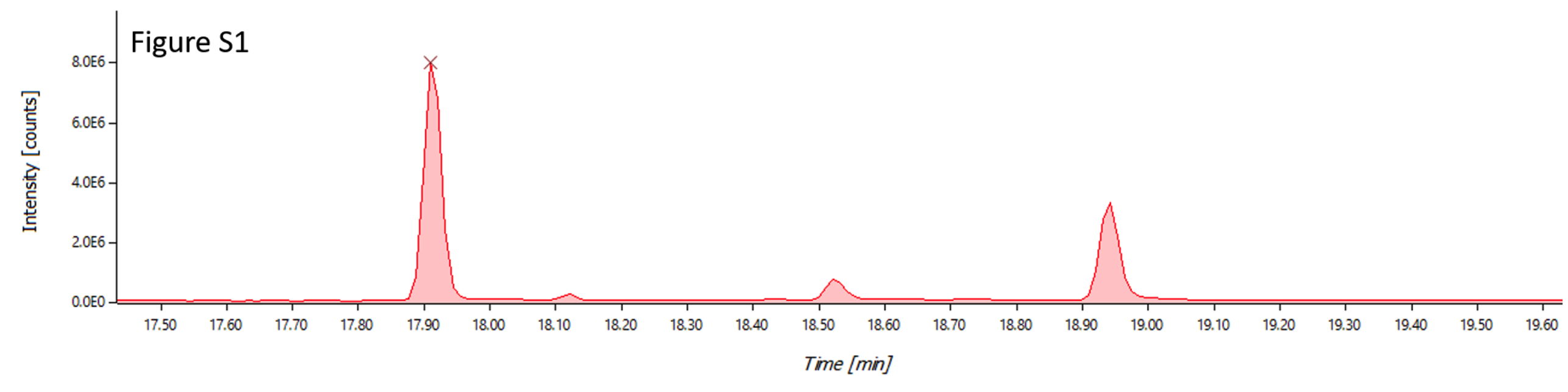
**

**Supplementary Figure 1.** Expanded lipid region of the chromatogram shown in Fig 5 showing two distinct lipid peaks.

**
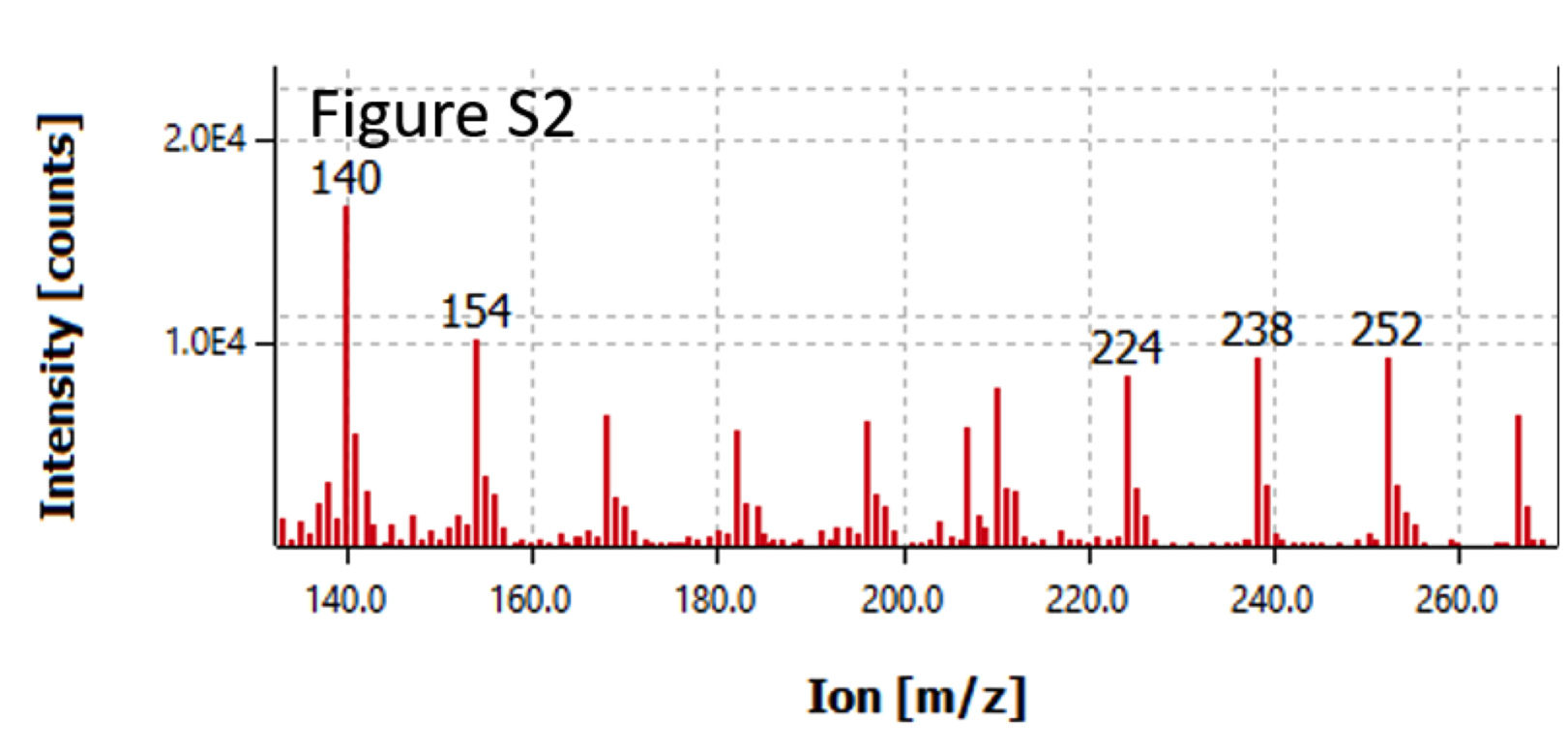
**

**Supplementary Figure 2.** MS of the peak in Fig S1 at 17.90 showing the step wise breakdown of 14 m/z. A strong indication of an acyl chain lipid.


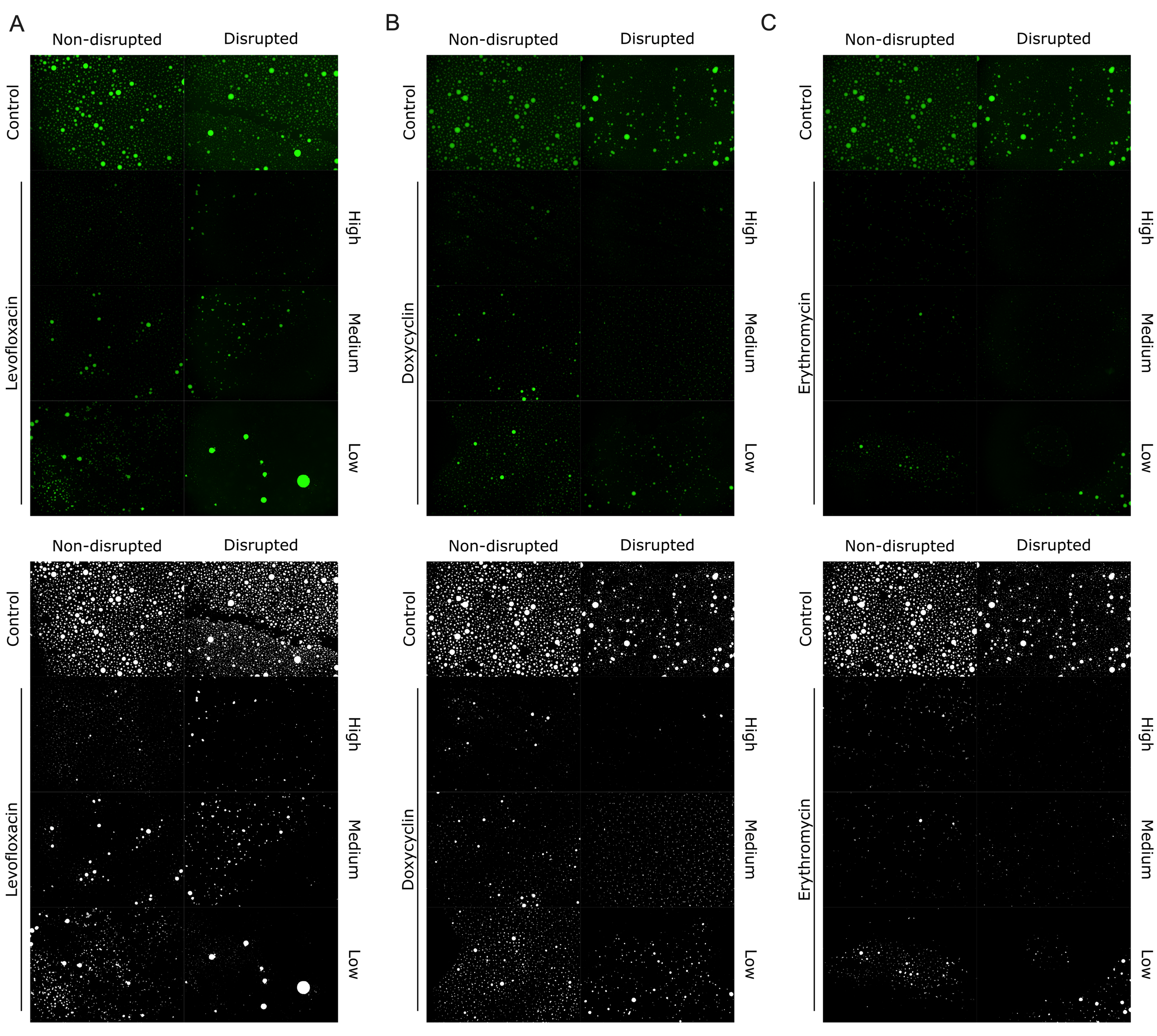


**Supplementary Figure 3.** Fluorescent confocal microscopic images of Mgen biofilms with and without antibiotic stress and disruption. Pre-formed biofilms were disrupted and then treated without or with antibiotics at high, medium or low concentrations. (A) Levo treatment and AF488-labeled HPG labeling. (B) Doxy treatment and AF488-labeled EdU labeling. (C) Ery treatment and AF488-labeled HPG labeling.
